# iMIRACLE: an Iterative Multi-View Graph Neural Network to Model Intercellular Gene Regulation from Spatial Transcriptomic Data

**DOI:** 10.1145/3627673.3679574

**Published:** 2024-10-21

**Authors:** Ziheng Duan, Siwei Xu, Cheyu Lee, Dylan Riffle, Jing Zhang

**Affiliations:** University of California, Irvine, Irvine, CA, USA; University of California, Irvine, Irvine, CA, USA; University of California, Irvine, Irvine, CA, USA; University of California, Irvine, Irvine, CA, USA; University of California, Irvine, Irvine, CA, USA

**Keywords:** inter-cellular gene regulation, cell–ell communications, spatial transcriptomics, graph neural networks

## Abstract

Spatial transcriptomics has transformed genomic research by measuring spatially resolved gene expressions, allowing us to investigate how cells adapt to their microenvironment via modulating their expressed genes. This essential process usually starts from cell-cell communication (CCC) via ligand-receptor (LR) interaction, leading to regulatory changes within the receiver cell. However, few methods were developed to connect them to provide biological insights into intercellular regulation. To fill this gap, we propose iMiracle, an iterative multi-view graph neural network that models each cell’s intercellular regulation with three key features. Firstly, iMiracle integrates inter- and intra-cellular networks to jointly estimate *cell-type*- and *micro-environment*-driven gene expressions. Optionally, it allows prior knowledge of intra-cellular networks as pre-structured masks to maintain biological relevance. Secondly, iMiracle employs iterative learning to overcome the sparsity of spatial transcriptomic data and gradually fill in the missing edges in the CCC network. Thirdly, iMiracle infers a cell-specific ligand-gene regulatory score based on the contributions of different LR pairs to interpret inter-cellular regulation. We applied iMiracle to nine simulated and eight real datasets from three sequencing platforms and demonstrated that iMiracle consistently outperformed ten methods in gene expression imputation and four methods in regulatory score inference. Lastly, we developed iMiracle as an open-source software and anticipate that it can be a powerful tool in decoding the complexities of inter-cellular transcriptional regulation.

## 1 Introduction

In eukaryotic organisms, precise spatial and temporal regulation of transcription is crucial for a range of fundamental biological processes, from development to adaptation to disease progression [6, 15, 16, 39–41, 57]. Thanks to concerted community efforts and technological advancements, there has been a remarkable leap over the past decades in our understanding of transcription regulation within individual cells [4, 7, 17, 23, 56, 59]. Thus, it has opened avenues for therapeutic strategies targeting specific transcriptional pathways and mechanisms [28]. While the current use of transcriptional technologies is promising, cells live in an organized combination of extracellular matrix, cells, and interstitial fluid that jointly influence gene expression [11, 46]. Aberrations in such intercellular communications within this spatial context may disrupt gene expression profiles, ultimately leading to cellular changes and pathogenic outcomes [24]. Despite its importance, the exploration of inter-cellular communication and its downstream impacts on transcriptional regulation remains underdeveloped. This gap limits our ability to fully understand multi-cellular functions and their implications for health and disease, highlighting an urgent need for new computational efforts.

To bridge this gap, we propose a novel, iterative multiview graph neural network (GNN) model named iMiracle to investigate intercellular transcriptional regulation for each cell. This model is designed with ***three distinct features*** to tackle the current challenges. First, iMiracle integrates inter- and intra-cellular networks for accurate expression imputation using ligand-receptor interactions with neighboring cells. Optionally, it allows users to include prior knowledge of intra-cellular networks, such as protein-protein interaction network (PPI) and gene regulatory network (GRN), as pre-structured masks to boost biological relevance [9]. Second, iMiracle employs iterative learning to gradually fill in the missing edges in the cell-cell communication (CCC) network, circumventing the limitations posed by the sparsity of spatial transcriptomic data. Lastly, iMiracle infers a cell-specific ligand-gene regulatory score based on the contributions of different LR pairs to interpret inter-cellular regulation.

We applied iMiracle to ***nine*** simulated and ***eight*** real datasets across three sequencing technologies for comprehensive performance benchmarking. We found that iMiracle consistently outperforms ten methods in the gene expression imputation task and four methods in the regulatory score inference task. Lastly, we developed iMiracle into an open-source software package ^1^ to facilitate its use by the scientific community for investigating inter-cellular transcriptional regulation at the individual cellular level. With the rapid expansion of spatial transcriptomics data, we anticipate that iMiracle will be a powerful tool in decoding the nuances of CCC in complex tissues, thus enriching our understanding of inter-cellular-level ligand-gene regulatory impacts.

## 2 Related Work

single-cell RNA sequencing (scRNA-seq) technology allows simultaneous gene expression profiling over thousands of cells, providing new opportunities to decipher inter-cellular transcriptional regulation [10, 21, 27, 42, 50]. Numerous methods have emerged to construct CCC networks based on ligand-receptor (LR) expression profiles [25]. While useful at their onset, they only focus on inter-cellular communication probabilities and do not delve into the transcriptional impacts on receiver cells. Later on, several methods were proposed to fill this gap by combining inter- and intra-cellular communications to link ligand genes from the sender cells directly to the target genes of the receiver cells. For example, NicheNet [3] combines inter-cellular CCC networks with prior knowledge of intra-cellular signaling and GRN to predict ligand-target gene regulatory scores. Cytotalk [60], on the other hand, combines cell-type-specific gene-co-expression networks with CCC networks to infer the regulatory potential of ligands on target genes. However, a challenge persists: scRNA-seq experiments require cell dissociation from their native tissue context, posing difficulties for accurate cell-specific inter-cellular regulatory relationship inference.

Current advancements in spatial transcriptomics have enabled spatially resolved gene expression profiling, enhancing our ability to explore transcription regulation within their native microenvironments [22, 32, 36]. Therefore, several computational methods were developed to utilize this new type of data. For instance, HoloNet [29] employed a multiview GNN to reconstruct gene expression and utilized an attention mechanism to calculate cell-type level ligand-gene regulatory score. However, the inherently sparse nature of spatial transcriptomics presents challenges in fully delineating the CCC network, resulting in an incomplete understanding of inter-cellular gene regulation [1]. Furthermore, it still lacks the granularity needed to explore ligand regulatory impacts at the level of individual cells.

## 3 Method

### 3.1 Method overview

**Figure 1:**
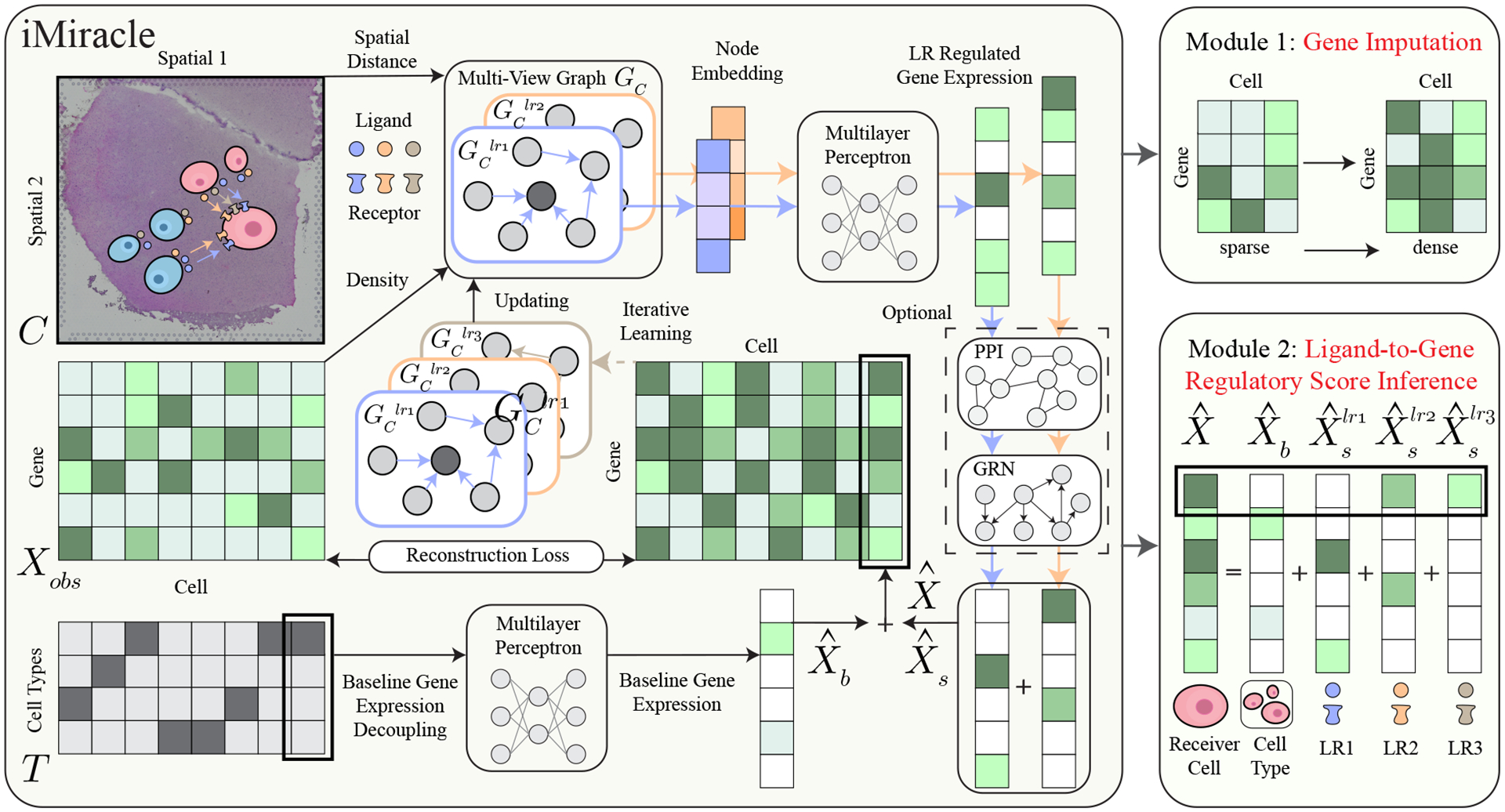
Overview of iMiracle. iMiracle initiates with a sparse cell-by-gene matrix $X_{obs}\in R^{n\times m}$ (n cells and m genes), spatial coordinates $C\in R^{n\times 2}$, and cell type information $T\in R^{n\times t}$ (t cell types). It constructs multi-view cell-cell communication networks ${\text{G}}_{\text{C}}$ to model various ligand-receptor interactions. Node embeddings for each cell are generated per view through a graph neural network. A multilayer perceptron then decodes gene expression ${\overset{ˆ}{X}}_{s}$ influenced by these LR interactions, integrating knowledge from an established gene regulatory network. iMiracle isolates the baseline gene expression matrix ${\overset{ˆ}{X}}_{b}$ solely determined by cell types. The final imputed gene expression matrix $\overset{ˆ}{X}$ merges the baseline matrix with expressions from ligand-receptor interactions. Through iterative learning, $\overset{ˆ}{X}$ is used to progressively refine the multi-view graph, enhancing both imputation precision and the inference of ligand-to-gene regulatory scores.

As shown in Fig. 1, our iMiracle model contains two key modules: 1) an iterative GNN for accurate ***gene expression imputation*** of individual cells using a multi-view CCC network among LR pairs; 2) cell-specific ***regulatory score inference*** from ligand genes (in sender cells) to target genes (in receiver cells). Formally, given the observed sparse cell-by-gene matrix $X_{\text{obs}}\in R^{n\times m}$ (n cells and m genes), the spatial coordinates $C\in R^{n\times 2}$, and the cell type information $T\in R^{n\times t}$ (t is the number of cell types), iMiracle imputes the dense gene expression matrix as $\overset{ˆ}{X}\in R^{n\times m}$ and provides a ranked list ${LR}_{\gamma }[c,g]$ to infer the regulatory score for cell c and gene g.

In its imputation module, iMiracle uniquely breaks down gene expression $\overset{ˆ}{X}$ into two distinct components: firstly, a cell-type-specific baseline expression ${\overset{ˆ}{X}}_{b}\in R^{n\times m}$, which is determined by the cell type, and secondly, a cell-specific expression ${\overset{ˆ}{X}}_{s}\in R^{n\times m}$, which is influenced by the micro-environment through CCC. As shown in Fig. 1, iMiracle integrates a multi-view inter-cellular CCC network with either a Multi-Layer Perceptron (MLP) or an optional pre-defined GRN/PPI to predict $\overset{ˆ}{X},\;{\overset{ˆ}{X}}_{b}$, and ${\overset{ˆ}{X}}_{s}$. Then, iMiracle iteratively updates the LR pairs based on the imputed gene expressions, repeating the estimation process until convergence. In its second module, iMiracle infers ligand-target gene regulatory score based on the contribution of each LR pair to a gene of interest in a cell-specific manner. We will introduce the model details in the following sections.

### 3.2 Module 1: Gene expression imputation via an iterative GNN

iMiracle imputes the gene expression matrix $\overset{ˆ}{X}$ without reference scRNA-seq data via three steps: constructing a multi-view CCC network, integrating inter- and intra-cellular networks, and iterative learning, as detailed below.

#### Multi-view CCC network construction.

As shown in Fig. 1, for each LR pair, we calculate the communication probability for each cell by synthesizing gene expression information and spatial distance, represented by $G_{C}^{lr}$. Then we combine all LR pairs’ CCC information via a multi-view network $G_{C}=\cup_{lr}G_{C}^{lr}$, where ∪ is the view aggregation. The CCC construction requires three steps:

Step 1: identify expressed LR pairs. Starting with LR pairs from CellChatDB [25] (3,267 pairs for humans and 3,387 for mice), we extract the expression level for ligand l and receptor r from $X_{\text{obs}}$. We define $S_{l}$ and $S_{r}$ as the sets of expression levels for l and r, respectively, and compute their geometric means as $E_{l}=gmean\left(S_{l}\right)$ and $E_{r}=gmean\left(S_{r}\right)$, each in $R^{n\times 1}$. Then proportions of expressed cells are: $\xi_{l}=\frac{1}{n}\sum_{i=1}^{n}\;1\left\{E_{l}[i]>0\right\}$ and $\xi_{r}=\frac{1}{n}\sum_{i=1}^{n}\;1\left\{E_{r}[i]>0\right\}$. One LR pair is considered expressed if both $\xi_{l}$ and $\xi_{r}$ exceed the predefined threshold θ (set at 15% by default), forming the set of biologically active LR pairs:
(1) $${LR}_{\theta }=\left\{lr|\xi_{l}>\theta \wedge \xi_{r}>\theta \right\}.$$
Step 2: calculate the distance for each cell. The Euclidean distance between cell c1 and cell c2 is calculated using their spatial coordinates (C[c1,:] and C[c2,:]). This results in the distance matrix $D\in R^{n\times n}$, capturing the spatial proximity of cells.
Step 3: compute the CCC network with gene expression. For each $\text{lr}\in {LR}_{\theta }$, the CCC network $G_{C}^{lr}$ is computed:
(2) $$G_{C}^{lr}=\left(E_{l}⊗E_{r}\right)⊙D^{-1}.$$
  The outer product ⊗ yields a matrix where each entry signifies the combined expression of l and r for each cell pair. Elementwise multiplication ⊙ merges this spatial data into the interaction strength assessment. Combining $G_{C}^{lr}$ for all LR pairs results in the multi-view CCC network $G_{C}$, enabling iMiracle to effectively model diverse CCC patterns.

#### Inter- and intra-cellular networks integration.

iMiracle integrates inter- and intra-cellular networks to infer gene expressions in individual cells. For each lr pair, the GNN outputs node embeddings to capture CCC’s impact fromlr as

(3) $$H_{lr}=GNN\left(T,G_{c}^{lr}\right),$$

where $H_{lr}\in R^{n\times d}$ is the d-dimensional node embedding inferred from the lr-specific GNN. To model the intra-cellular regulation, iMiracle transforms $H_{lr}$ into a gene expression matrix for each lr pair, followed by a shared decoder:

(4) $${\overset{ˆ}{X}}_{s}^{lr}=Decoder\left(H_{lr}\right).$$

Here ${\overset{ˆ}{X}}_{s}^{lr}\in R^{n\times m}$ reflects the gene expression regulated by the specific lr interactions. The decoder, typically implemented as an MLP, is designed to map each cell’s embedding to its gene expression vector. Optionally, iMiracle can integrate the pre-structured GRN/PPI via:

(5) $${\overset{ˆ}{X}}_{s}^{lr^{\prime }}={\overset{ˆ}{X}}_{s}^{lr}⊙M_{lr},$$

where $M_{lr}\in R^{m}$ is a binary mask derived from the GRN/PPI, with ones representing possible regulation and zeros otherwise. In addition, iMiracle uses an MLP to capture baseline gene expression profiles that are solely influenced by cell type:

(6) $${\overset{ˆ}{X}}_{b}=MLP\left(T\right).$$

Then iMiracle synthesizes the cell-type-specific and cell-specific expression as the final gene expression $\overset{ˆ}{X}$:

(7) $$\overset{ˆ}{X}={\overset{ˆ}{X}}_{b}+\sum_{lr\in {LR}_{\theta }}{\overset{ˆ}{X}}_{s}^{lr^{\prime }}.$$

#### Iterative learning.

Spatial transcriptomic data usually has excessive missing values in $X_{\text{obs}}$, leading to incomplete CCC estimation and thus limiting the imputation performance. To address this issue, iMiracle employs iterative learning to gradually refine the multi-view graph $G_{C}$ based on the imputed expression matrix $\overset{ˆ}{X}$. Specifically, after the i-th training iteration, ${LR}_{\theta }^{\left(i+1\right)}$ is updated as:

(8) $${LR}_{\theta }^{\left(i+1\right)}=\left\{lr|\xi_{l}^{\left(i+1\right)}>\theta \wedge \xi_{r}^{\left(i+1\right)}>\theta \right\}.$$

Here $\xi_{l}^{\left(i+1\right)}$ and $\xi_{r}^{\left(i+1\right)}$ represent the updated proportions of expressed cells, which are computed using the updated ${\overset{ˆ}{X}}^{\left(i+1\right)}$. We next update the CCC network for each LR pair that exists in both $LR_{\theta }^{\left(i\right)}$ and ${LR}_{\theta }^{\left(i+1\right)}$. Combining previous CCC network ${G_{c}^{lr}}^{\left(i\right)},{G_{c}^{lr}}^{\left(i+1\right)}$ is updated as:

(9) $$G_{c}^{lr^{\left(i+1\right)}}=\alpha G_{c}^{lr^{\left(i\right)}}+\left(1-\alpha \right)G_{c}^{lr^{\left(i+1\right)}}.$$

A blending coefficient α harmonizes the contributions from both old and new estimates to ensure a smooth update. For LR pairs in ${LR}_{\theta }^{\left(i+1\right)}$ but not in the ${LR}_{\theta }^{\left(i\right)}$, they directly form new CCC networks: $G_{c}^{lr^{\left(i+1\right)}}$, which is derived from ${\overset{ˆ}{X}}^{\left(i+1\right)}$. Merging existing and newly added CCC networks, we have:

(10) $$G_{C}^{\left(i+1\right)}=\cup_{lr\in {LR}_{\theta }^{\left(i+1\right)}}{G_{c}^{lr}}^{\left(i+1\right)}.$$

#### Model training and hyperparameter tuning.

During the training phase, iMiracle aims to minimize the Mean Squared Error (MSE) between $\overset{ˆ}{X}$ and $X_{\text{obs}}$. The loss function is particularly focused on non-zero entries of $X_{\text{obs}}$:

(11) $$ℒ=\frac{\sum_{i=1}^{n}\;\sum_{j=1}^{m}\;1\left[X_{obs,(i,j)}\neq 0\right]{\left({\overset{ˆ}{X}}_{\left(i,j\right)}-X_{obs,(i,j)}\right)}^{2}}{\sum_{i=1}^{n}\;\sum_{j=1}^{m}\;1\left[X_{obs,(i,j)}\neq 0\right]},$$

where 1[⋅] is an indicator that equals 1 if $X_{obs,(i,j)}\neq 0$ and 0 otherwise. We will stop the iteration if no new views can be added, as it suggests a saturation in constructing a full CCC.

We developed iMiracle using PyTorch version 1.12.1, operational on an Nvidia GeForce RTX A6000 GPU. Our computational setup is powered by an AMD EPYC 7302 16-Core Processor (1.0 TiB of memory) and operates on the Ubuntu 20.04.1 LTS system. In the gene imputation process, if there’s no decrease for 10 consecutive epochs, we terminate the training and proceed to evaluate whether there’s a need to update views. For the ligand-target gene regulatory score inference, training is halted if the loss fails to reduce by more than 0.001 over 10 successive epochs, after which we assess the necessity of updating views. For both tasks, we set the hidden dimension d to 32, the blending coefficient α to 0.2, the number of neighbors k to 5 for graph construction, a default two GNN layers, the maximum number of epochs to 1000, and use a learning rate of 0.01 with the Adam optimizer (details in the parameter analysis).

### 3.3 Module 2: Cell-specific regulatory score inference

After training, iMiracle aims to identify ligands (in sender cells) that significantly impact gene expression (in receiver cells). For a specific cell c and gene g, the lr-related regulatory score ψ(lr,c,g) is defined as:

(12) $$\psi (lr,c,g)={\overset{ˆ}{X}}_{s}^{lr^{\prime }}[c,g].$$

Here ${\overset{ˆ}{X}}_{s}^{lr^{\prime }}[c,g]$ represents the lr-regulated strength for cell c and gene g. Based on ψ(lr,c,g), iMiracle evaluates lr pairs within ${LR}_{\theta }$ and gives a ranked list:

(13) $${LR}_{\gamma }\left[c,g\right]=\text{sort}\left[lr\in {LR}_{\theta },\psi \left(lr,c,g\right)\;\text{descending}\;\text{order}\right].$$

${LR}_{\gamma }[c,g]$, ordered by regulatory score, enables iMiracle to pinpoint key LR pairs affecting gene regulation in individual cells, offering insights into inter-cellular regulation dynamics.

### 3.4 Simulation details

Following [29], we created simulated data, which includes 1000 cells in a 100-unit square space, for benchmarking. We assigned cell types based on their locations (using a parameter $k_{b}$ that controls the mixing of cell types) and modeled gene expression for 50 genes (using a negative binomial distribution with high: $\left(n_{h},p_{h}\right)$ and low: $\left(n_{l},p_{l}\right)$). To simulate CCC, 50 LR pairs were selected, with specific high-expression areas (a radius of r units and $\left(n_{c},p_{c}\right)$) designated for intensified interactions. Gene expressions were updated to reflect these selected LR interactions. Next, we randomly masked the simulated data, maintaining a density of 20% to reflect the spatial data’s sparsity. To ensure fairness, we designed nine different settings (Table 1) and reported performance across varied settings.

### 3.5 Data preprocessing and experimental setup

#### Preprocessing details.

We include human dorsolateral prefrontal cortex (DLPFC) datasets from 10X Visium platform [35], mouse olfactory bulb dataset from Steroseq [5], and mouse olfactory bulb dataset from SlideseqV2 [48]. We follow pre-processing steps as suggested in the original paper (a summary can be seen in Table 2). Detailed methodologies for preprocessing and obtaining PPI and GRN are shown in the appendix.

#### Benchmark baselines and evaluation metrics.

For the gene imputation task, data is down-sampled with 10% of non-zero entries allocated for testing and another 10% for validation [53]. To ensure fairness, this procedure is repeated ten times, each with different mask configurations. Imputed gene expressions are compared to ground truth using L1 Distance, Root-Mean-Square Error (RMSE), and Cosine Similarity. We evaluate ten leading methods, including scRNA-seq data analysis tools like scVI [34], ALRA [31], eSNN [49], MAGIC [51], and scGNN [53], which overlook spatial information. Additionally, gimVI [33] and Tangram [2], capable of integrating reference scRNA-seq, are tested in a reference-free mode for fairness. Spatial transcriptomics-specific methods like seSNN [43], STLearn [37], and STAGATE [8] are included.

For ligand-gene regulatory score inference using simulated data, we employed four evaluation metrics: Precision, Normalized Discounted Cumulative Gain (NDCG), Spearman Correlation, and Kendall Rank Correlation. Our comparison includes NicheNet [3], SpaTalk [44], and HoloNet [29], assessing their ability to rank LR pairs based on their influence on specific genes within cells, with a random guess approach as a naive baseline. We use default settings for all baseline methods.

## 4 Results

### 4.1 iMiracle delineates the full landscape of CCC network via iterative learning

**Figure 2:**
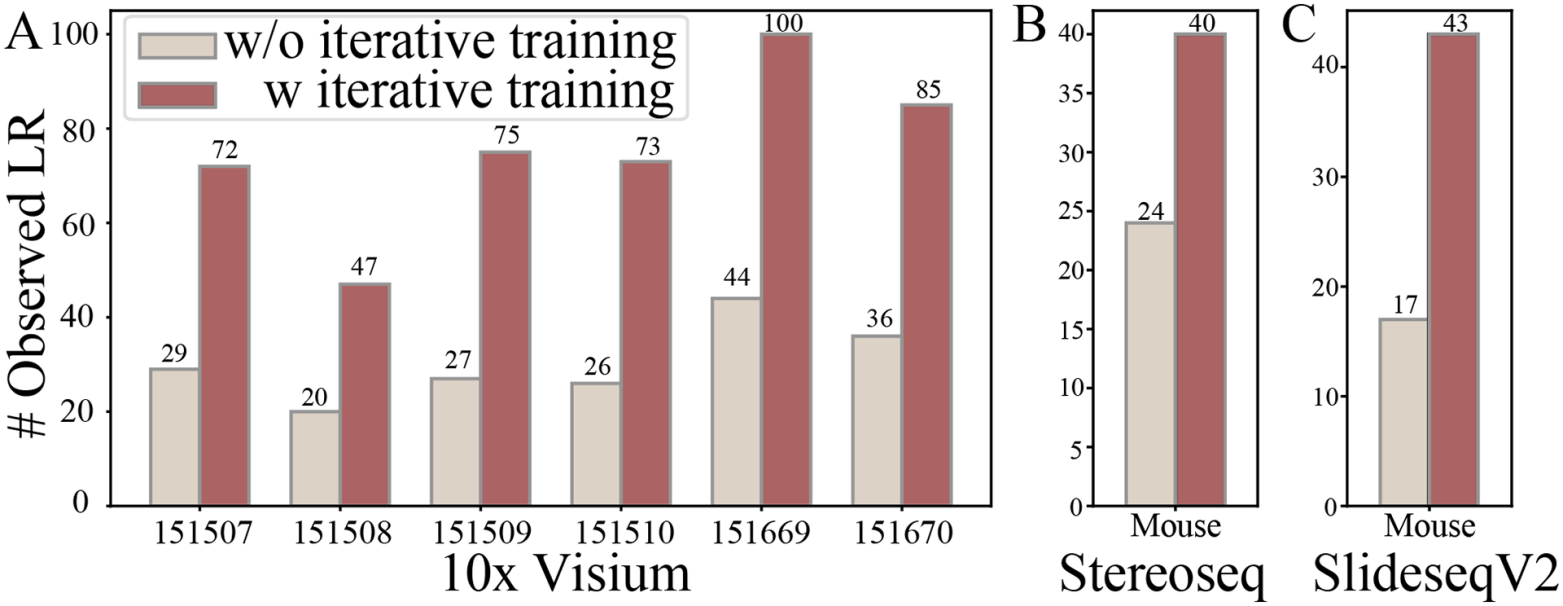
iMiracle fully delineates the CCC network via iterative learning, uncovering up to 181% more LR interactions in 10x Visium, 67% in Stereoseq, and 153% in SlideseqV2.

To test the efficacy of iterative learning, we evaluated its role in the gradual delineation of the full landscape of CCC on eight datasets. Specifically, we compared the number of views in the constructed CCC, in other words, the number of included LR pairs. We found that iMiracle’s iterative learning process noticeably increased the LR pairs included in $G_{C}$. For instance, on the 10x Visium datasets, iMiracle identified an increase of 27 to 56 LR pairs across six samples in the final iteration compared to the first round (Fig. 2A). This trend was consistent across all sequencing platforms, with an addition of 16 LR pairs in Stereoseq (Fig. 2B) and 26 in SlideseqV2 (Fig. 2C). The increased LR pair information enriched the spatial information in the GNN, potentially facilitating the downstream expression imputation and regulatory score inference tasks.

### 4.2 iMiracle consistently boosts imputation accuracy on diverse datasets

Next, we evaluated iMiracle’s imputation performance against ten recent methods on diverse real datasets across three popular platforms (10x Visium, Stereoseq, and SlideseqV2) and two species (human and mouse). Due to the lack of gold standard benchmark datasets, we down-sampled the observed data and used the masked values as the ground truth to calculate three metrics, including L1 Distance, RMSE, and Cosine Similarity.

As shown in Table 3, iMiracle notably outperformed the best spatially-informed methods and demonstrated even larger improvements when compared to the top scRNA-seq-based baselines. On the SlideseqV2 dataset, for example, iMiracle achieved a 47% RMSE improvement over STAGATE, the foremost spatial method, and a 52% RMSE improvement over scGNN, the top non-spatial method. Specifically, among all methods utilizing cell coordinates, GNN-based approaches, such as STAGATE and iMiracle, demonstrated superior performance, supported by an average RMSE improvement of 66% over other spatial techniques. In addition, iMiracle exhibited higher imputation accuracy than STAGATE (RMSE 0.407 vs 0.765), attributable to its iterative learning and the multi-view network that combines both gene expression and distance information, as opposed to STAGATE’s single-view GNN architecture derived mainly from the spatial distance. We also tested other datasets and found that iMiracle consistently reported the best gene imputation accuracy in all three metrics, indicating the robustness of our method across diverse sequencing platforms.

### 4.3 iMiracle highlights accurate inter-cellular ligand-gene regulatory insights

**Figure 3:**
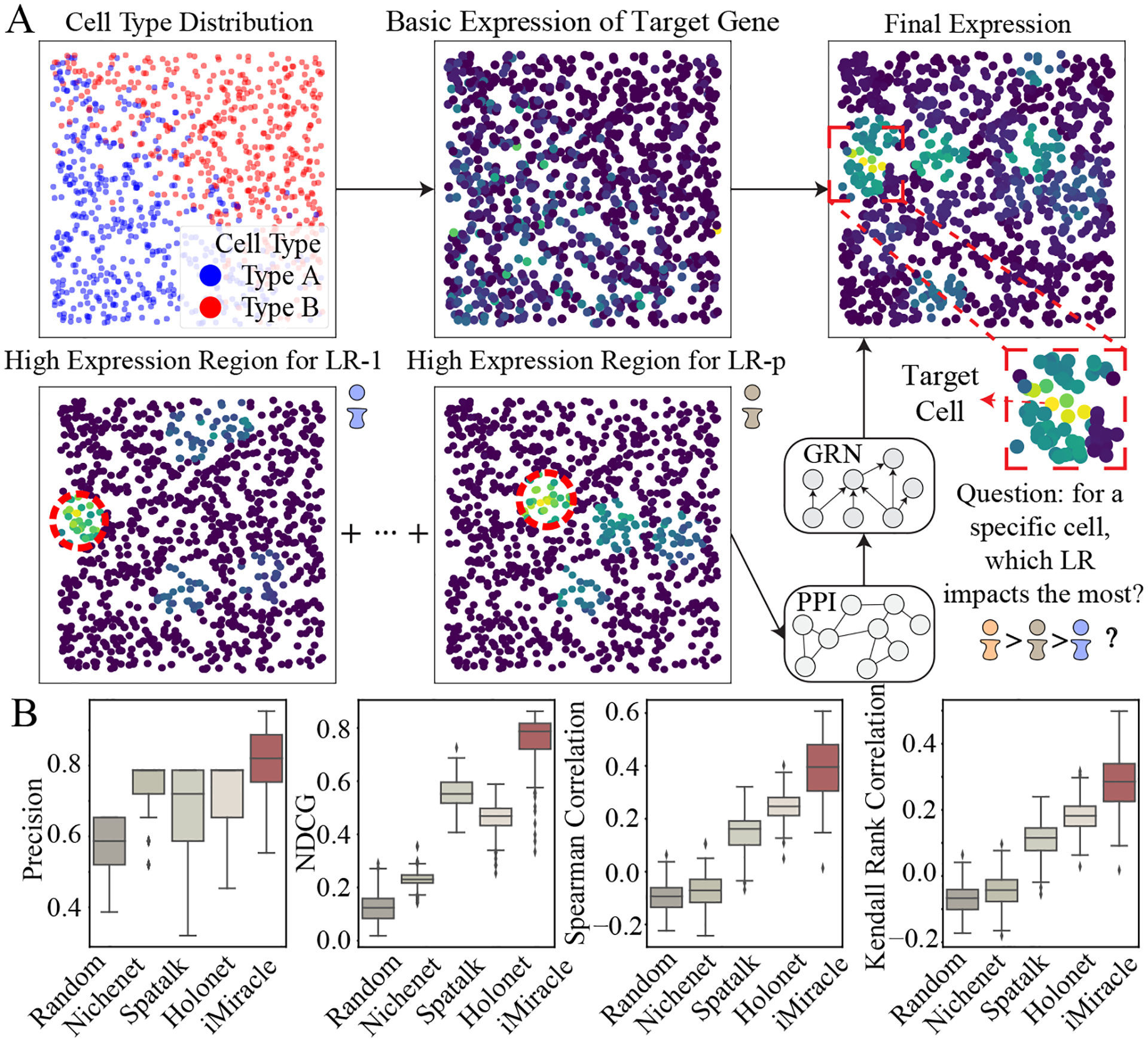
iMiracle consistently outperforms other methods in the regulatory score inference. (A) In this simulation, 1000 cells are spatially arranged in a 100-unit square. Cell types were determined by their locations, incorporating high-expression zones for various LR pairs, to realistically model gene expression and CCC dynamics. This setup is utilized for inferring cellular-level regulatory scores. (B) Benchmarking results demonstrate iMiracle’s superior accuracy in inferring ligand-gene regulatory scores, surpassing all four baselines across all four metrics.

We benchmarked iMiracle with four other methods in terms of their ability to accurately capture ligand-target gene regulatory relationships across various simulated datasets (Fig. 3A, details see methods). Using known ligand-gene score as ground truth, we found that iMiracle consistently outperformed all the other methods (Fig. 3B). For instance, iMiracle demonstrated a noticeable improvement in NDCG (0.79 vs 0.24, Fig. 3B) when compared to NichNet, a gain largely due to its effective integration of spatial information. Among the spatial methods, iMiracle stood out as the best, surpassing SpaTalk and HoloNet (NDCG 0.79 vs 0.56/0.48, Fig. 3B). This trend was not only evident in NDCG but also consistent across other metrics such as precision, Spearman Correlations, and Kendall Rank Correlations. Such consistent performance highlights the benefit of using iterative learning to comprehensively map the CCC network, as well as its integration of both inter- and intra-cellular networks. This approach provides a more detailed, cell-specific view of cellular communication. Furthermore, iMiracle’s improved performance was affirmed under various parameter settings (details in the appendix), underscoring the model’s adaptability and effectiveness in diverse research contexts.

### 4.4 iMiracle reveals substantial regulatory heterogeneity across cells of the same type

One unique advantage of iMiracle is its ability to split gene expression into separate components driven by cell-type and the microenvironment, offering vital insights into how ligands differentially influence target genes within a specific spatial context. Therefore, our approach can quantitatively assess cell-specific spatial impacts of inter-cellular regulation and reveal regulatory variations among cells of the same type. We demonstrated this via a case study by estimating each LR’s regulatory score to *GJA1*, a canonical marker gene in Astrocytes with essential functions in gap junction formation and DLPFC functionality [38, 47].

**Figure 4:**
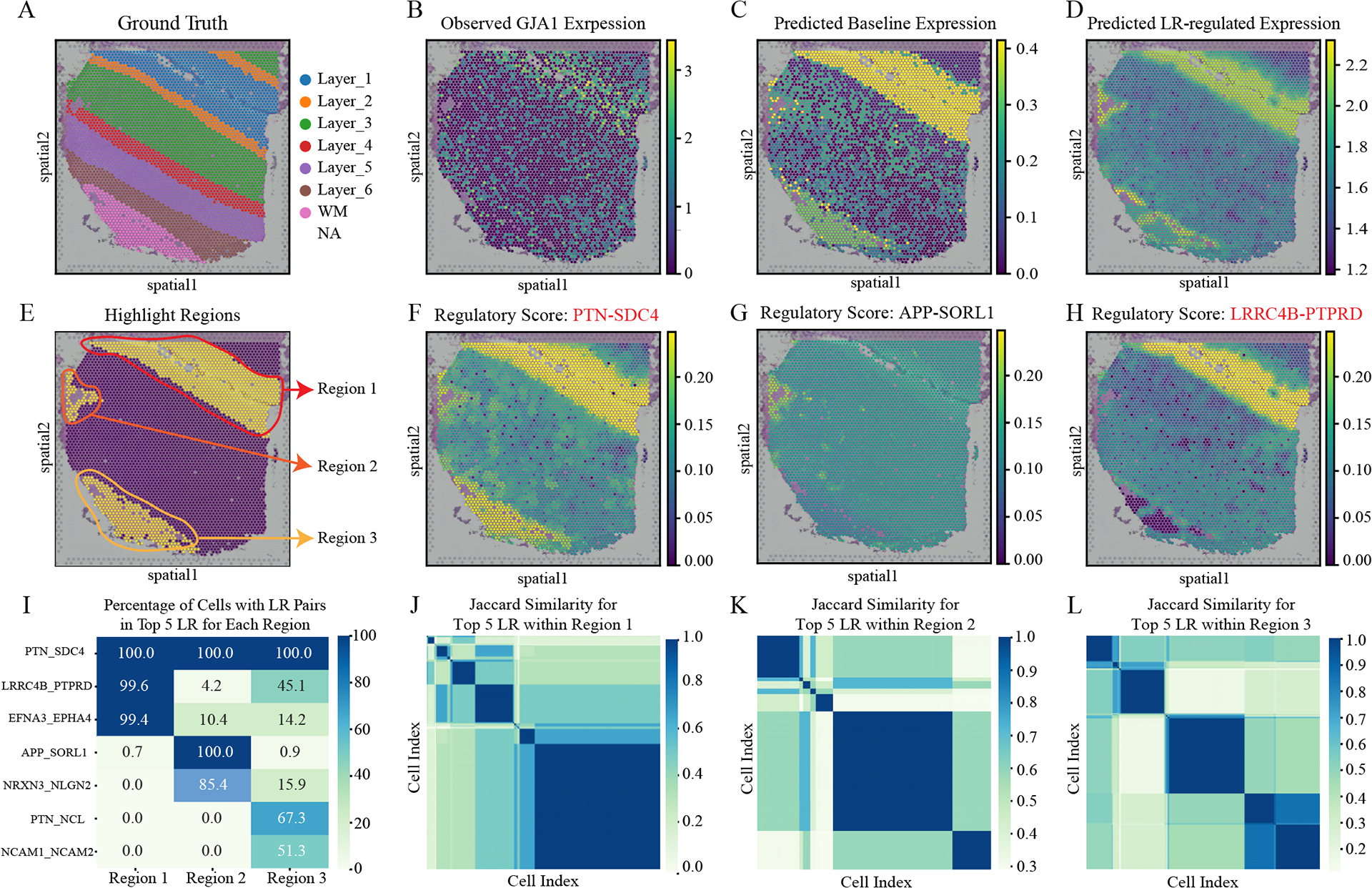
iMiracle reveals substantial regulatory heterogeneity across cells. (A) Detailed ground truth segmentation of the cortical layers and white matter (WM) within the DLPFC section of sample 151507. (B) Visualization of the observed expression pattern of *GJA1*. (C) Prediction of the baseline expression profile for *GJA1*. (D) Prediction of the LR-regulated expression for *GJA1*. (E) Identification of three key regions within sample 151507. (F-H) Top three LR interactions and their corresponding regulatory scores. LR pairs *PTN-SDC4* and *LRRC4B-PTPRD* were discovered through an iterative learning approach, indicated by their red colors. (I) Heatmap illustration of the percentage of cells featuring the top five LR pairs in each identified region. (J-L) *Jaccard similarity* of the top five LR pairs for cells within each region, revealing substantial regulatory heterogeneity across cells.

Specifically, we identified regions with high LR regulatory scores and gene expression of *GJA1* and intersected them with different layers, resulting in three unique regions to begin with (Fig. 4A&E). The top three LR pairs with the highest average regulatory scores were selected: *PTN-SDC4*, *APP-SORL1*, and *LRRC4B-PTPRD*. It’s noteworthy that two out of three LR pairs (*PTN-SDC4* and *LRRC4B-PTPRD*) were identified via iterative learning, highlighting the importance of relying on dense imputed data.

We first compared the observed expression patterns with the two predicted components from iMiracle: cell-type-driven and micro-environment-driven expressions (Fig. 4B-D). These patterns showed high consistency in our visualizations. When we analyzed the cell-specific regulatory scores, we noticed substantial heterogeneity and distinct patterns for different LR pairs. For instance, the *PTN-SDC4* pair exhibited consistent scores across all regions, whereas the *APP-SORL1* and *LRRC4B-PTPRD* pairs showed strong preferences in specific regions (Fig. 4F-H). This finding underscores the importance of including cell-specific contexts in modeling processes, as relying solely on average cell-type-specific scores would obscure such significant regulatory diversity.

Finally, we compared both cross-region and within-region regulatory heterogeneity of the top 5 LR pairs. Only one LR pair *PTN-SDC4* was consistent across all three regions, while the remaining ones were highly regional-specific (Fig. 4I). For instance, *LRRC4B-PTPRD* pair ranked among the top 5 LR pairs in 99.6% of cells in region 1, whereas it was present in only 4.2% and 45.1% of cells in regions 2 and 3, respectively. Next, we looked at the regulatory heterogeneity within each region. Specifically, we calculated the *Jaccard similarity* of the identified top 5 LR pairs among cells within each region, as shown in Fig. 4J-L. Similarly, distinct LR usage preferences were discovered among cells within all three selected regions, demonstrating the pressing need to account for each cell’s micro-environment when characterizing inter-cellular transcription regulation.

### 4.5 Ablation study to evaluate the effectiveness of iMiracle’s modeling components

To assess each component of our model, we performed a variant analysis, considering four different versions: 1) “w/o GRN”, which excludes the integration of prior biological knowledge; 2) “w/o iterations”, a straightforward, non-iterative approach using sparse gene expressions; 3) “shared GNN”, where the same GNN parameters are applied to all ligand-receptor (LR) pairs; and 4) “view decoder”, implementing a unique decoder for each LR pair. This analysis allowed us to isolate and understand the individual contribution of each component to the overall performance of the model.

**Figure 5:**
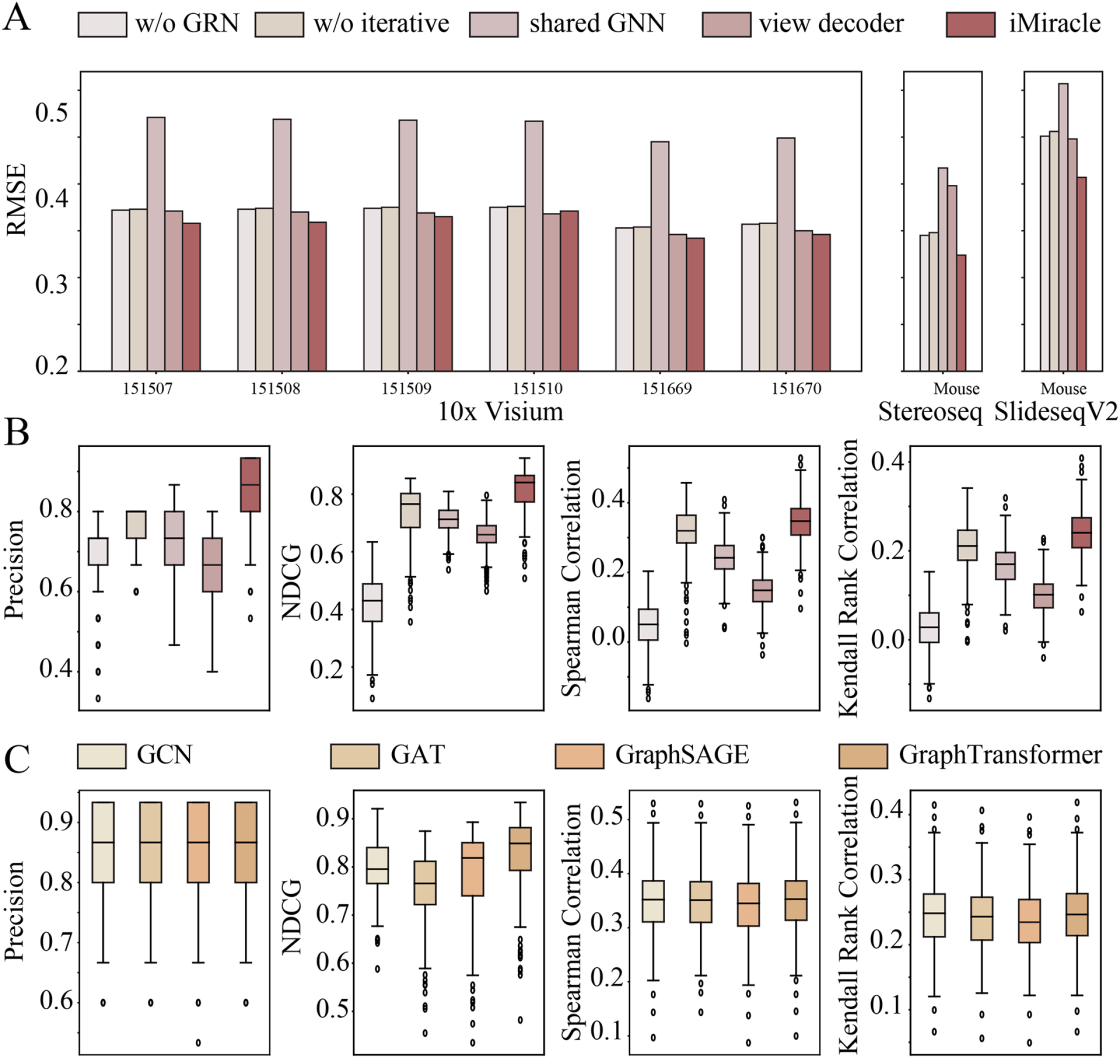
Variant analysis. (A) RMSE w.r.t. different variants of iMiracle for gene imputation. (B) Four regulatory score inference metrics w.r.t. variants of iMiracle. (C) Four regulatory score inference metrics w.r.t. GNN architectures.

Firstly, after removing prior knowledge of intra-cellular network, we observed a slight decrease in gene imputation accuracy (1–11%, Fig. 5A) and a more pronounced reduction in regulatory score inference (NDCG: 0.43 vs 0.84, Fig. 5B). This outcome underscores the critical role of integrating biological knowledge for generating biologically meaningful interpretations. Next, the non-iterative model variant exhibited a slightly reduced accuracy in the regulatory score inference (NDCG: 0.78 vs 0.84, Fig. 5B), indicating the advantages of adopting an iterative approach. Then we found that employing shared GNN parameters led to a significant decline in gene imputation performance (25–32%, Fig. 5A), highlighting the necessity for diverse message propagation strategies across different views in $G_{C}$. Lastly, using view-specific decoders adversely affected regulatory performance (NDCG: 0.66 vs 0.84, Fig. 5B), pre-sumably due to the increased complexity in training arising from a higher number of parameters.

We also tested iMiracle’s adaptability to different GNN architectures using GCN [26], GAT [52], GraphSAGE [20], and Graph-Transformer [45]. Results showed comparable performance across these architectures (Fig. 5C), demonstrating iMiracle’s flexibility and efficacy with various GNN models. We use GraphTransformer as our default setting.

### 4.6 Parameter analysis

**Figure 6:**
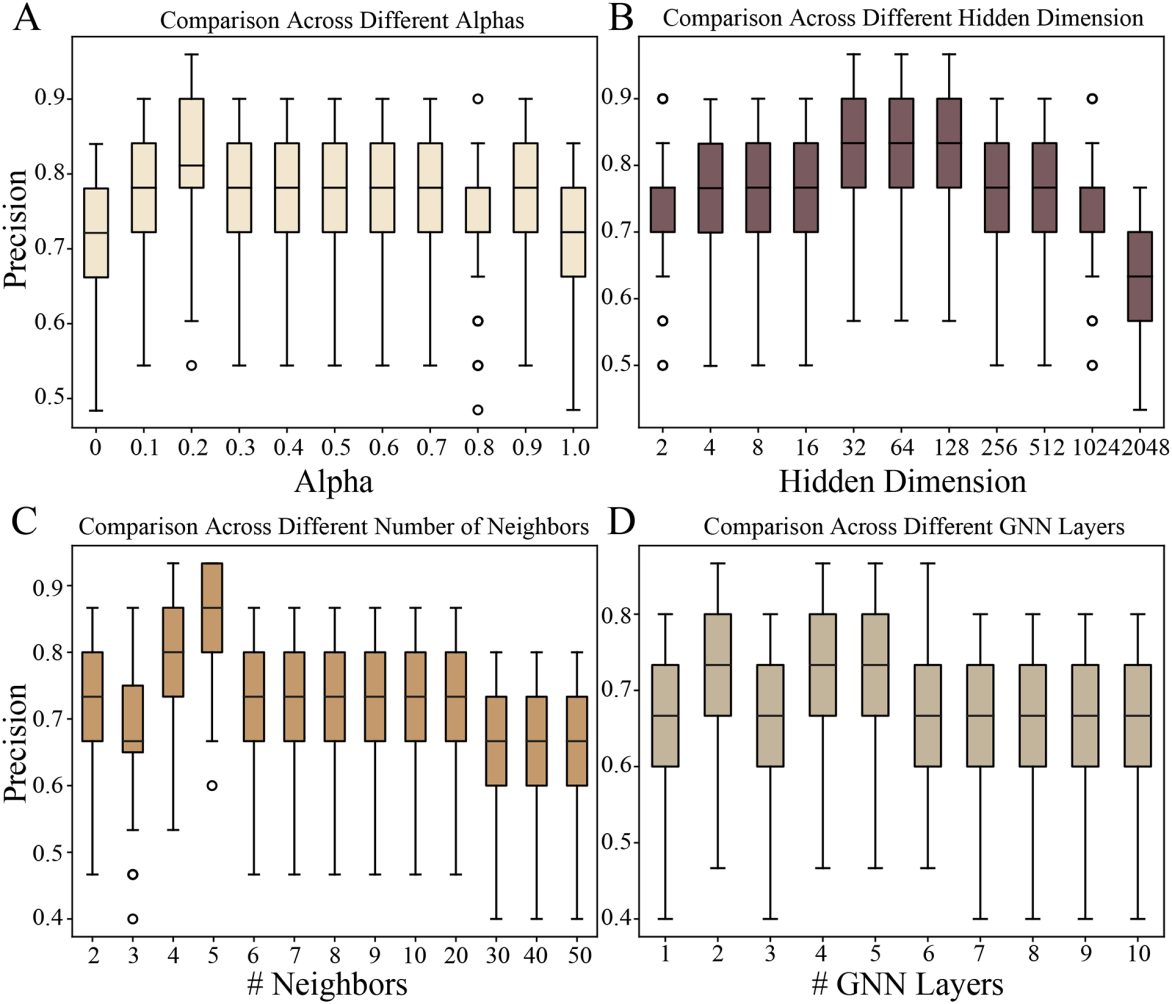
Parameter analysis. (A) Precision w.r.t. different blending coefficient α. (B) Precision w.r.t. different hidden dimension d. (B) Precision w.r.t. different number of neighbors k for graph construction. (B) Precision w.r.t. GNN layers L.

To showcase the robustness of iMiracle in response to varying parameters, we employed the simulated data to assess its precision over a broad spectrum of blending coefficients α, hidden dimensions d, the number of neighbors k for graph construction, and the GNN layers L [12–14, 54, 55, 58]. As depicted in Fig. 6A, an α value of zero indicates exclusive reliance on newly imputed gene expressions for determining the existing graph structure. Conversely, an α value of one signifies maintaining the original graph structure of existing views. Both extremes lead to a reduction in precision. An α value of 0.2 results in optimal performance, underscoring the importance of smoothly integrating updated gene expression profiles into the multi-view graph. Exploring a wide range of hidden dimensions d, from 2 to 2048, we observed that iMiracle demonstrates considerable robustness in regulatory score inference, except at extreme values (i.e., 2, 1024, or 2048) from Fig. 6B. We choose 32 as the default d. Also, we set the number of neighbors k=5 for graph construction, and the GNN layers L=2 for optimal balance between performance and complexity as shown in Fig. 6C-D.

## 5 Conclusion and Discussion

In our study, we introduce iMiracle, a novel computational tool tailored for spatial transcriptomic data, aiming to unravel the complexities of inter-cellular transcriptional regulation. Unlike conventional methods that offer only averaged ligand regulatory scores across diverse micro-environments, iMiracle uniquely identifies the effects of gene expression caused by neighboring cells using CCC, separating these from effects due to the inherent characteristics of the cell type itself. This distinction enables iMiracle to investigate regulatory dynamics with unparalleled precision for a deeper understanding of inter-cellular transcriptional regulation.

iMiracle distinguishes itself from existing approaches via three key features designed explicitly for spatial transcriptomic data. Firstly, it integrates spatial distance and LR expression profiles to construct a multi-view inter-cellular CCC network, offering more biologically relevant insights with greater depth of information than methods mainly based on spatial distance (e.g., STAGATE). This integration, especially when combined with prior knowledge of intra-cellular networks (such as GRNs and PPIs), allows for more accurate and interpretable gene expression imputation, a benefit confirmed through extensive benchmarking on various datasets (Table 3). Secondly, iMiracle utilizes iterative learning to progressively refine the CCC network, effectively addressing data sparsity and uncovering more impactful LR pairs, as shown in our analyses on several real datasets (Fig. 2). Finally, it excels in inferring cell-specific ligand-gene regulatory scores, a feature often over-looked in approaches that neglect micro-environment effects (Fig. 3). We demonstrated the evident benefit of this feature by reporting substantial regulatory heterogeneity in cells under different spatial contexts (Fig. 4). A minor concern regarding iMiracle is that it necessitates a cell-by-cell-type (or spot-by-cell-type-proportion) matrix as input to estimate baseline cell-type-specific gene expression. As a result, inaccuracies in cell type assignment or cell proportion calculation could affect the imputation performance. However, the impact of such inaccuracies is likely to be mitigated by ongoing and future advancements in spatial resolution and sequencing depth in technologies.

iMiracle has been developed as an open-source software freely available for researchers exploring inter-cellular gene expression regulation at the individual cellular level. Given the rapid advancements in spatial transcriptomics and the increasing availability of public data, iMiracle may serve as an essential tool in unraveling the complexities of cell-to-cell communication networks in complex tissues, thereby enriching our understanding of inter-cellular transcriptional regulation dynamics across various biological contexts.

## Figures and Tables

**Table 1:** Summary of simulation data parameters.

ID	*k*_*b*_	*r*	(*n*_*h*_, *p*_*h*_)	(*n*_*l*_, *p*_*l*_)	(*n*_*c*_, *p*_*c*_)
1	*2*	10	(8, 0.5)	(2, 0.8)	(4, 0.8)
2	*2*	10	(8, 0.5)	(2, 0.8)	(8, 0.8)
3	*5*	10	(8, 0.5)	(2, 0.8)	(4, 0.8)
4	*5*	10	(8, 0.5)	(2, 0.8)	(8, 0.8)
5	*5*	10	(8, 0.5)	(4, 0.8)	(4, 0.8)
6	*5*	10	(8, 0.5)	(4, 0.8)	(8, 0.8)
7	*5*	20	(8, 0.5)	(2, 0.8)	(4, 0.8)
8	10	10	(8, 0.5)	(2, 0.8)	(8, 0.8)
9	20	10	(8, 0.5)	(2, 0.8)	(8, 0.8)

**Table 2:** Summary of real datasets.

Platform	Organism	Sample ID	Raw Matrix (Cell, Gene)	Raw Density	Filter Matrix (Cell, Gene)	Filter Density	# Imputed Entries
10xVisium	Human Dorsolateral Prefrontal Cortex (DLPFC)	151507	4226, 33538	0.042	4147, 4028	0.262	437240
		151508	4384, 33538	0.036	4148, 3342	0.258	358184
		151509	4789, 33538	0.043	4700, 4188	0.258	508186
		151510	4643, 33538	0.041	4547, 3908	0.259	461112
		151669	3661, 33538	0.054	3617, 5246	0.277	525930
		151670	3498, 33538	0.050	3433, 4909	0.272	457770
Stereoseq	Mouse	/	19109, 14376	0.024	4036, 1581	0.193	123444
SlideseqV2	Mouse	/	20139, 11750	0.031	5161, 2611	0.217	292418

**Table 3:** Gene imputation benchmark.

Metric	Method		Platform & Dataset							
			10xVisium						Stereoseq	SlideseqV2
			DLPFC						Mouse	Mouse
			151507	151508	151509	151510	151669	151670	/	/
L1 Distance	w/o	scVI	0.794±0.004	0.838±0.006	0.800±0.002	0.670±0.003	0.810±0.003	0.696±0.005	1.442±0.005	1.127±0.006
		ALRA	0.499±0.003	0.512±0.001	0.490±0.001	0.496±0.001	0.467±0.002	0.472±0.002	0.406±0.013	0.649±0.066
		eSNN	1.254±0.001	1.373±0.001	1.266±0.001	1.294±0.000	1.017±0.001	1.071±0.001	2.802±0.002	2.071±0.002
		Magic	0.779±0.001	0.825±0.001	0.787±0.000	0.664±0.001	0.795±0.001	0.692±0.000	1.324±0.001	1.080±0.000
		scGNN	0.583±0.011	0.665±0.085	0.589±0.011	0.584±0.004	0.550±0.006	0.532±0.009	0.819±0.240	0.664±0.018
	w	gimVI	0.838±0.003	0.890±0.003	0.835±0.001	0.737±0.002	0.863±0.003	0.765±0.001	1.325±0.001	1.153±0.002
		seSNN	1.254±0.001	1.371±0.001	1.266±0.000	1.294±0.000	1.017±0.001	1.072±0.001	2.775±0.002	1.998±0.001
		Tangram	1.691±0.001	1.811±0.001	1.689±0.000	1.420±0.000	1.728±0.001	1.474±0.000	2.899±0.001	2.185±0.000
		STLearn	1.333±0.001	1.423±0.001	1.332±0.001	1.148±0.001	1.369±0.002	1.206±0.001.	NA	NA
		STAGATE	0.297±0.001	0.300±0.002	0.295±0.005	0.294±0.004	0.274±0.005	0.278±0.002	0.289±0.006	0.502±0.007
		iMiracle	**0.271**±**0.001**	**0.280**±**0.001**	**0.271**±**0.002**	**0.272**±**0.001**	**0.263**±**0.001**	**0.265**±**0.002**	**0.203**±**0.003**	**0.284**±**0.004**
Cosine Similarity	w/o	scVI	0.907±0.001	0.913±0.001	0.906±0.001	0.903±0.001	0.909±0.001	0.904±0.001	0.941±0.001	0.919±0.002
		ALRA	0.948±0.002	0.952±0.002	0.952±0.001	0.952±0.001	0.938±0.006	0.944±0.003	0.980±0.002	0.927±0.018
		eSNN	0.842±0.000	0.841±0.000	0.839±0.000	0.840±0.000	0.846±0.000	0.843±0.000	0.777±0.001	0.838±0.000
		Magic	0.915±0.000	0.920±0.000	0.914±0.000	0.909±0.000	0.916±0.000	0.910±0.000	0.968±0.002	0.936±0.000
		scGNN	0.933±0.004	0.927±0.016	0.932±0.002	0.936±0.000	0.917±0.002	0.929±0.002	0.948±0.035	0.953±0.002
	w	gimVI	0.957±0.000	0.965±0.001	0.955±0.001	0.947±0.001	0.962±0.001	0.948±0.002	0.964±0.000	0.936±0.001
		seSNN	0.843±0.000	0.841±0.000	0.840±0.000	0.841±0.000	0.851±0.000	0.847±0.000	0.768±0.000	0.817±0.000
		Tangram	0.713±0.001	0.725±0.001	0.717±0.001	0.716±0.001	0.717±0.001	0.715±0.000	0.772±0.001	0.763±0.001
		STLearn	0.718±0.000	0.718±0.000	0.715±0.001	0.724±0.000	0.715±0.001	0.717±0.000	NA	NA
		STAGATE	0.983±0.000	0.985±0.000	0.983±0.001	0.984±0.001	0.980±0.001	0.980±0.000	0.990±0.000	0.961±0.000
		iMiracle	**0.985**±**0.000**	**0.987**±**0.000**	**0.985**±**0.000**	**0.985**±**0.000**	**0.982**±**0.001**	**0.982**±**0.000**	**0.996**±**0.000**	**0.990**±**0.000**
RMSE	w/o	scVI	0.940±0.005	0.993±0.006	0.949±0.003	0.803±0.003	0.959±0.003	0.834±0.005	1.628±0.005	1.307±0.007
		ALRA	0.784±0.003	0.810±0.005	0.766±0.001	0.777±0.001	0.735±0.004	0.743±0.003	0.723±0.036	1.061±0.107
		eSNN	1.378±0.001	1.503±0.000	1.393±0.000	1.419±0.001	1.143±0.002	1.199±0.001	2.778±0.001	2.177±0.001
		Magic	0.917±0.001	0.972±0.001	0.929±0.000	0.792±0.000	0.936±0.001	0.824±0.000	1.453±0.001	1.238±0.001
		scGNN	0.755±0.016	0.850±0.096	0.762±0.011	0.755±0.002	0.717±0.007	0.686±0.010	1.051±0.307	0.842±0.021
	w	gimVI	0.955±0.002	1.002±0.001	0.957±0.001	0.858±0.001	0.970±0.002	0.890±0.002	1.448±0.001	1.217±0.004
		seSNN	1.354±0.001	1.474±0.000	1.370±0.000	1.395±0.001	1.119±0.001	1.175±0.001	2.770±0.002	2.087±0.001
		Tangram	1.768±0.001	1.889±0.001	1.767±0.000	1.503±0.000	1.804±0.001	1.557±0.000	2.970±0.001	2.284±0.000
		STLearn	1.516±0.001	1.629±0.001	1.521±0.001	1.300±0.001	1.556±0.002	1.362±0.001	NA	NA
		STAGATE	0.384±0.002	0.393±0.002	0.379±0.007	0.380±0.007	0.357±0.007	0.365±0.004	0.485±0.008	0.765±0.005
		iMiracle	**0.358**±**0.000**	**0.359**±**0.001**	**0.365**±**0.001**	**0.371**±**0.001**	**0.342**±**0.000**	**0.346**±**0.001**	**0.324**±**0.003**	**0.407**±**0.007**

The best results are bolded. Results marked ‘NA’ for stLearn indicate unavailable HE stained images required by the method. “w/o” and “w” mean methods without and with spatial information, respectively.

**Figure 7:**
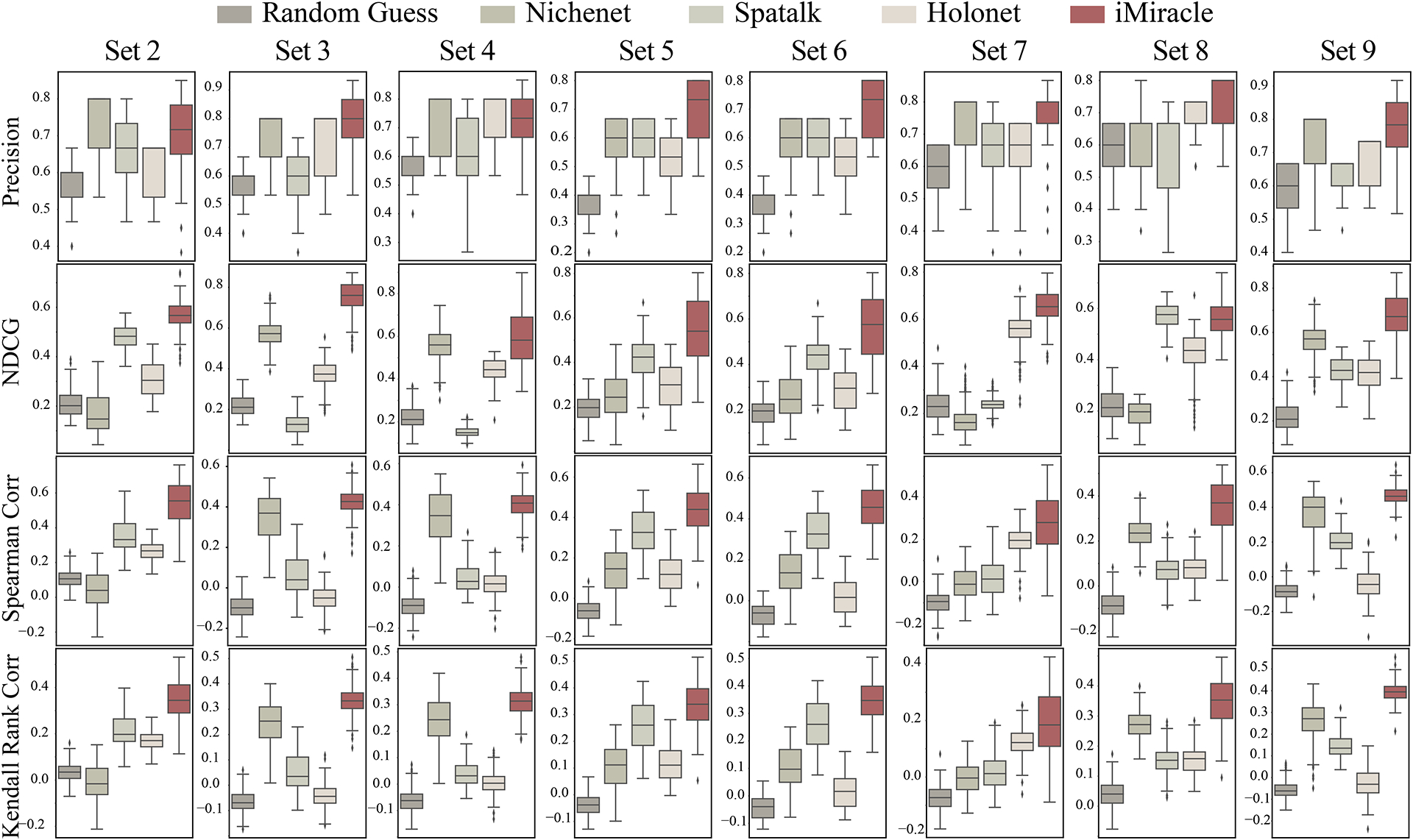
Additional regulatory score inference results.

## CCS Concepts

• Applied computing → Bioinformatics; • Computing methodologies → Machine learning approaches.

## Footnotes

1
https://github.com/aicb-ZhangLabs/iMiracle

## A Data Preprocessing Details

For real-world data preprocessing, we first filtered the cells and genes for quality assurance. Only cells with at least 500 detected genes and genes expressed in at least 10% of cells were retained. Next, we normalized the total counts per cell to a target sum of 1e4 and applied a log transformation to the data.

The protein-protein interaction (PPI) was obtained from a previous study named InWeb published in 2016 [30]. Specifically, we used the InWeb_InBioMap PPI table for Homo Sapiens which contained a total of 18,814 genes (vertices) and 883,356 interactions (edges). For the cell type-specific GRN in the brain prefrontal cortex region, we used a data processing pipeline from a set of private single-cell multiome data that contain scRNA-seq and scATAC-seq modalities. We first conducted a cellranger-arc call and basic QC to create the curated scRNA-seq matrix and the scATAC-seq fragment. Then, we used ArchR (v1.0.1) [19] to do cell type-specific peak calling and peak-to-gene interaction with functions addReproduciblePeakSet() and addPeak2GeneLinks(). In the peak calling step, we used Macs2 (version 2.2.7.1) [61] and did not limit the maximum number of peaks per cell type. In the peak-to-gene linkage creation step, we used the LSI created with 30 dimensions and used a correlation cutoff of 0.45 and a resolution of 500,000bp upstream and downstream. Then, we retrieve the motif-to-peak correspondence (motif annotation) from JASPAR2020 [18]. Using the annotation and the created peak-to-gene linkage, we construct the motif-to-gene graph if any peak connects to a gene and a motif.

To establish the mask from ligand to target gene, we processed genes associated with specific cell types and LR pairs. For each LR pair, genes were identified and expanded using PPI data to include associated genes. Subsequently, we integrated this information with GRN data to identify regulatory genes corresponding to each LR pair for different cell types. This process resulted in a comprehensive mapping, effectively linking LR pairs to their regulatory target genes, and thereby capturing the complex interplay within cellular communication networks.

## B Complexity Analysis

We explored the complexity of iMiracle from two aspects: its time complexity, determined by various computational steps, and its parameter count, influenced by the distinct components.

### B.1 Time Complexity Analysis of iMiracle

The time complexity of iMiracle is composed of four key steps: the basic gene expression decoupling, the multi-view graph construction, lr-specific GNNs, and the shared decoder. The basic MLP, processing cell type and gene expression data, incurs a complexity of O(n×(t×d+d×m)). For the multi-view graph construction, each adjacency matrix $A_{lr}$ computation entails a complexity of $O\left(n^{2}\right)$. With p unique LR pairs, the total complexity for this component is $O\left(p\times n^{2}\right)$. The computational load for each GNN layer is O(|E|×d), where |E| denotes the number of edges in the sparse adjacency matrix. For all p LR pairs, this accumulates to O(p×|E|×d). The decoders, applied to the embeddings from each lr-specific GNN, involve matrix operations resulting in a complexity of O(p×n×(d×d+d×m)). Considering t≪m and d≪m, the overall time complexity of iMiracle can be summarized as $O\left(p\times n^{2}+p\times d\times (|E|+n\times m)\right)$.

### B.2 The Number of Parameters in iMiracle

iMiracle’s parameter complexity is influenced by its basic MLP, lr-specific GNNs, and decoders. The basic MLP comprises parameters of O(t×d+d×m). For each lr-specific GNN, the parameter count is O(t×d+d×d). With p unique LR pairs, the total parameters across all GNNs amount to p×O(t×d+d×d). Similarly, the shared decoder contributes an additional O(d×d+d×m) parameters. Given that t≪m and d≪m, the overall parameter count of iMiracle is O(d×(p×(t+d)+m)).

## C Additional Performance Analysis

We benchmarked iMiracle with four other methods in terms of their ability to accurately capture ligand-target gene regulatory relationships under other eight simulation settings. As shown in Fig. 7, iMiracle consistently achieves the best performance across different settings and evaluation metrics.
